# Characterization and Antibacterial Potential of Lactic Acid Bacterium *Pediococcus pentosaceus* 4I1 Isolated from Freshwater Fish *Zacco koreanus*

**DOI:** 10.3389/fmicb.2016.02037

**Published:** 2016-12-20

**Authors:** Vivek K. Bajpai, Jeong-Ho Han, Irfan A. Rather, Chanseo Park, Jeongheui Lim, Woon Kee Paek, Jong Sung Lee, Jung-In Yoon, Yong-Ha Park

**Affiliations:** ^1^Department of Applied Microbiology and Biotechnology, Yeungnam UniversityGyeongsan, South Korea; ^2^National Science Museum, Ministry of Science, ICT and Future PlanningDaejeon, South Korea; ^3^KcellbioSeoul, South Korea

**Keywords:** *Pediococcus pentosaceus* 4I1, *Zacco koreanus*, foodborne pathogens, antimicrobial action, cell free supernatant

## Abstract

This study was undertaken to characterize a lactic acid bacterium 4I1, isolated from the freshwater fish, *Zacco koreanus*. Morphological, biochemical, and molecular characterization of 4I1 revealed it to be *Pediococcus pentosaceus* 4I1. The cell free supernatant (CFS) of *P. pentosaceus* 4I1 exhibited significant (*p* < 0.05) antibacterial effects (inhibition zone diameters: 16.5–20.4 mm) against tested foodborne pathogenic bacteria with MIC and MBC values of 250–500 and 500–1,000 μg/mL, respectively. Further, antibacterial action of CFS of *P. pentosaceus* 4I1 against two selected bacteria *Staphylococcus aureus* KCTC-1621 and *Escherichia coli* O157:H7 was determined in subsequent assays. The CFS of *P. pentosaceus* 4I1 revealed its antibacterial action against *S. aureus* KCTC-1621 and *E. coli* O157:H7 on membrane integrity as confirmed by a reduction in cell viability, increased potassium ion release (900 and 800 mmol/L), reduced absorption at 260-nm (3.99 and 3.77 OD), and increased relative electrical conductivity (9.9 and 9.7%), respectively. Gas chromatography–mass spectrometry (GC–MS) analysis of the CFS of *P. pentosaceus* 4I1 resulted in the identification of seven major compounds, which included amino acids, fatty acids and organic acids. Scanning electron microscopic-based morphological analysis further confirmed the antibacterial effect of CFS of *P. pentosaceus* 4I1 against *S. aureus* KCTC-1621 and *E. coli* O157:H7. In addition, the CFS of *P. Pentosaceus* 4I1 displayed potent inhibitory effects on biofilms formation by *S. aureus* KCTC-1621 and *E. coli* O157:H7. The study indicates the CFS of *P. pentosaceus* 4I1 offers an alternative means of controlling foodborne pathogens.

## Introduction

Lactic acid bacteria (LAB) have proven efficacies for food preservation and enhance the nutritive quality of a variety of fermented food products (Gad et al., 2016). Primarily, the LAB exert their antimicrobial effects by producing lactic acid, which increases the acidity of their environment, thereby resulting in the loss of the viabilities of pathogenic bacteria (Ammor et al., 2006; Gad et al., 2016). Low molecular weight compounds, such as, hydrogen peroxide, carbon dioxide, diacetyl (2,3-butanedione), and bacteriocins also contribute to antimicrobial effects of LAB (Ammor et al., 2006; Sankarankutty and Palav, 2016), as they substantially inhibit the growths of pathogenic bacteria in food system. Nowadays, consumers show considerable interest in the use of LAB as natural additives in food industry due to their generally recognized as safe (GRAS) and antimicrobial effects (Parada et al., 2007; Djadouni and Kihal, 2012).

Recently, severe concerns have been expressed regarding the increasing incidences of diseases associated with foodborne pathogens (Oussalah et al., 2007; da Silveira et al., 2012; Gad et al., 2016). Although adequate biopreservation techniques can prevent food contamination by pathogens, food processors are frequently confronted by situations involving food contamination by pathogenic microorganisms (Runyoro et al., 2010). In addition, the increased resistance shown by pathogenic microbes to commercial antibiotics (Manzoor et al., 2016) has generated much interest in the identification of new classes of natural antibiotics that are capable of combating hazardous pathogens (Millitello et al., 2011).

Recent reports confirm wide consumer acceptance of the use of natural preservatives in foods and the need to reduce the amounts of synthetic additives used (Millitello et al., 2011; da Silveira et al., 2012). Although synthetic additives have been used to retard the growths of foodborne pathogens, they pose serious threats to human health (Carocho et al., 2015). On the other hand, LAB diminish foodborne pathogen proliferation and offer consumers safer and contamination free food products (Callewaert et al., 2000; Mataragas et al., 2003). Furthermore, a number of studies have demonstrated the remarkable efficacies of LAB to control foodborne pathogens *in vitro* and *in vivo* (Callewaert et al., 2000; Mataragas et al., 2003; Ammor et al., 2006; Manzoor et al., 2016).

The present study was undertaken to isolate and characterize a lactic acid bacterium *Pediococcus pentosaceus* 4I1 from *Zacco koreanus*, a freshwater fish, and to examine its antibacterial mechanistic action on selected foodborne pathogens.

## Materials and Methods

### Media, Reagents, and Test Sample Preparation

Bromocresol purple (BCP) agar medium was used for the culture-based initial screening of LAB, whereas nutrient broth (NB) medium was used to cultivate pathogenic bacteria. Both media were purchased from Sigma-Aldrich (Sigma, St. Louis, MO, USA). The de Man, Rogosa, and Sharpe (MRS) agar medium used to isolate and culture LAB was purchased from Difco (USA). Other chemicals and reagents used were of high purity. Spectrophotometric measurements were made using an enzyme-linked immunosorbent assay (ELISA) instrument. A 18∼24 h grown culture of *P. pentosaceus* 4I1 was centrifuged followed by freeze-drying and desired test concentrations of its cell free supernatant (CFS) were prepared in double-distilled sterilized water.

### Foodborne Pathogens

The foodborne pathogens tested were *Salmonella enterica* ATCC-4731, *Staphylococcus aureus* KCTC-1621, *Listeria monocytogenes* KCTC-3569, *Bacillus subtilis* KCTC-3569, and *Escherichia coli* O157:H7. Test pathogens were cultured in NB medium and incubated at 37°C. For regular experiments, cultures were maintained on nutrient agar (NA) medium and stored at 4°C.

### Sample Collection and Isolation of *P. pentosaceus* 4I1

*Zacco koreanus* specimens were collected from a local river using catch per unit effort (CPUE) methods following taxonomic identification (Kim and Park, 2002) and isolation procedure of LAB (Cho et al., 2013). Initial screening was performed using the agar spot test on BCP agar medium (Trias et al., 2008). Selected LAB working cultures were then routinely grown in MRS broth and stored as stock cultures in MRS broth containing 15% glycerol (cryoprotective agent) in cryovials at -20°C. Ethical approval regarding “Animal Care and Use” was secured beforehand from the ethical committee of Daejeon National Science Museum Daejeon, South Korea with an approval # NSMD-MSIFP-KOR208. Experiments were performed in accordance with the relevant guidelines and methods.

### Morphological, Biochemical, and Molecular Characterization of 4I1

Morphological, biochemical, and molecular characterizations of 4I1 were performed as we previously described (Bajpai et al., 2016a). In brief, 4I1 was identified by gram-staining and based on microscopic observations of colony shapes and cell morphologies (Holt et al., 1994). Further, 4I1 was characterized biochemically using API 50 CHL strips with API 50 CHL medium at the species level according to the manufacturer’s instructions (API 50 CHL, BioMerieux, France).

Partial 16S rRNA gene sequencing analysis was used to characterize 4I1 at the molecular level. Briefly, genomic DNA was isolated from 4I1 and then the 16S rRNA gene was amplified by PCR (Bajpai et al., 2016b). PCR reactions were carried out using a Biometra thermal cycler (M Biotech, Inc., Canada) with the following cycle parameters: an initial denaturation at 94°C for 2 min, followed by 35 cycles of denaturation at 94°C for 30 s, annealing at 52°C for 30 s, and elongation at 72°C for 1 min. The PCR products were sequenced and analyzed, and gene sequences obtained were compared in the National Center for Biotechnology Information (NCBI) for homology using BLAST and multiple-aligned with 16S rRNA gene sequences of different strains for similarity using the ClustalW program coupled with MEGA 5. The neighbor-joining method was used to construct the phylogenic tree using MEGA 5 software.

### Gas Chromatography–Mass Spectrometry (GC–MS) Analysis

A detailed analysis of the chemical composition of the CFS of *P. pentosaceus* 4I1 was performed as described by Sjögren et al. (2003) using a gas chromatography–mass spectrometry (GC/MS) system (Jeol JMS 700 mass spectrometer, Agilent 6890N, Agilent Technologies, Santa Clara, CA, USA) equipped with a fused silica capillary column (30 m length × 0.25 mm ID × 0.25 μm film thickness). The GC–MS conditions used were as previously described (Bajpai et al., 2013). Relative proportions of the extract constituents were expressed as percentages by peak area normalization. Extract components were identified based on GC retention times and computer matching of mass spectra using the Wiley and National Institute of Standards and Technology Libraries for the GC–MS system used.

### Determination of the Effect of 4I1 CFS on Antibacterial Activity

The standard agar well-diffusion method was used to determine the antibacterial efficacy of 4I1 CFS (Murray et al., 1995). To obtain the CFS of 4I1, supernatant from a 24 h grown culture of 4I1 was collected by centrifugation (8,000 × *g*; 10 min) and freeze-dried (lyophilized) (Saadatzadeh et al., 2013). Briefly, NA medium (20 mL) was poured into Petri-plates and allowed to solidify. Plates were then dried and 1 mL of standardized bacterial inoculum (10^7^ CFU/mL) was poured and uniformly spread onto agar surfaces, and then allowed to stand for 5 min. Wells were made in the agar by using a sterilized borer and 100 μL CFS of *P. pentosaceus* 4I1 was poured into each well against each of the tested pathogen. Negative controls were prepared using the same medium (sterilized distilled water or MRS medium) employed to dissolve the samples. Antibacterial activities were evaluated by measuring the diameters of zones of inhibition (including diameter of well: 6 mm) against the tested bacteria. All assays were performed in triplicate.

### Determination of the Effect of 4I1 CFS on Minimum Inhibitory (MIC) and Minimum Bactericidal (MBC) Concentrations

The MICs of CFS of *P. pentosaceus* 4I1 were determined using the twofold serial dilution method (Bajpai et al., 2013). Freeze-dried CFS of *P. pentosaceus* 4I1 (4 mg) was first dissolved in 1 mL distilled water as stock, and incorporated into NB medium to an initial concentration of 2,000 μg/mL, and then was serially diluted to 1,000, 500, 250, 125, 62.5, 31.25, 15.62, and 7.81 μg/mL concentrations of the CFS of 4I1. A 10 μL standardized bacterial suspension of each tested pathogen (10^7^ CFU/mL) was transferred to each tube. The treatment and control tubes which contained only bacterial suspensions were incubated at 37°C for 24 h. The lowest concentration of CFS, which did not show any visible growth of tested organisms after macroscopic evaluation, was determined as MIC, and was expressed in μg/mL. Further, the concentrations showing complete inhibition of visual growth of bacterial pathogens were identified, and 50 μL of each culture broth was transferred onto the agar plates and incubated at 37°C for 24 h. The complete absence of growth of bacterial colonies on the agar surface is the lowest concentration of the sample and was defined as MBC. Each assay in this experiment was replicated three times.

### Determination of the Effect of 4I1 CFS on Pathogen Viabilities

Freshly grown bacterial colonies of the selected pathogenic bacteria were inoculated in NB medium at 37°C for 24 h, and then bacterial cultures were serially diluted to 10^7^ CFU/mL (Shin et al., 2007). To determine the effect of CFS of *P. pentosaceus* 4I1 on cell viabilities, two selected foodborne pathogenic bacteria, *S. aureus* KCTC-1621 and *E. coli* O157:H7 were used. Briefly, each of the tubes containing bacterial suspension (10 μL; approximately 10^7^ CFU/mL) of *S. aureus* KCTC-1621 and *E. coli* O157:H7 was inoculated with 100 μL of CFS of 4I1 at its MIC in 890 μL NB broth at 37°C. Samples for viable cell counts were taken out at 0, 40, 80, 120, 160, and 200 min time intervals. Viable plate counts were monitored on NB agar as we previously described (Bajpai et al., 2013). Colonies were counted after incubation for 24 h at 37°C. The controls were inoculated without CFS of 4I1 for each pathogenic bacteria using the same experimental condition. Assay were performed in triplicate.

### Determination of the Effect of 4I1 CFS on Potassium Ion Efflux

The effects of CFS of *P. pentosaceus* 4I1 on the efflux of potassium ion from *S. aureus* KCTC-1621 and *E. coli* O157:H7 were determined as we previously described (Bajpai et al., 2013). Concentration of free potassium ion in bacterial suspensions of *S. aureus* KCTC-1621 and *E. coli* O157:H7 was measured after exposing bacterial cells to CFS of *P. pentosaceus* 4I1 at their MICs in sterile peptone water (8.5 g NaCl + 1 g peptone in 1 L sterilized distilled water) for 0, 30, 60, 90, and 120 min. At each pre-established interval, the extracellular potassium concentration was measured by a photometric procedure using the Calcium/Potassium kit (Quantofix, GmbH, Wiesbaden, Germany). Similarly, control was also tested without adding CFS. Results were expressed as the amount of extracellular free potassium (mmol/L) in the growth media in each interval of incubation.

### Determination of the Effect of 4I1 CFS on the Release of 260-nm Absorbing Materials

The release of 260-nm-absorbing materials from *S. aureus* KCTC-1621 and *E. coli* O157:H7 cells was monitored in aliquots of 2 mL of the bacterial inocula in sterile peptone water (0.1 g/100 mL). The reaction solution containing MIC of CFS of *P. pentosaceus* 4I1 was incubated at 37°C. At 0, 30, and 60 min time interval of treatment, cells were centrifuged at 3,500 × *g*, and the absorbance of the obtained supernatant was measured at 260 nm using a 96-well plate ELISA reader (Bajpai et al., 2013). Controls were treated in the same manner without CFS of *P. pentosaceus* 4I1. Results were expressed in terms of optical density (OD) of 260-nm absorbing materials in each interval with respect to the ultimate time.

### Determination of the Effect of 4I1 CFS on Cell Membrane Permeability

The effects of CFS of *P. pentosaceus* 4I1 on cell membrane permeability of *S. aureus* KCTC-1621 and *E. coli* O157:H7 were determined as described previously (Patra et al., 2015), and expressed in terms of relative electrical conductivity. Upon action of CFS on the cell membrane of tested pathogens, it may cause drastic release of cytosolic materials such as protein, DNA and other essential metabolites, resulting in the cell death. Prior to the assay, cultures of test pathogens were incubated at 37°C for 10 h, followed by centrifugation (5,000 × *g*) for 10 min, and washed with 5% glucose solution (w/v) until their electrical conductivities reached close to 5% glucose solution to induce an isotonic condition. MICs of CFS of *P. pentosaceus* 4I1 acquired for both the tested pathogens were added to 5% glucose (isotonic solution), incubated at 37°C for 8 h, and the electrical conductivities (L_a_) of the reaction mixtures were determined. Further, electrical conductivities of the bacterial solutions were measured at 2 h of intervals for a total duration of 8 h (L_b_). The electrical conductivity of each test pathogen in isotonic solution killed by boiling water for 5 min served as a control (L_c_). The relative electrical conductivity was measured using an electrical conductivity meter. The permeability of bacterial membrane was calculated according to the following formula:

$$\text{Relative conductivity }\left(\%\right)\,={\text{L}}_{\text{a}}\,\;-\;{\text{L}}_{\text{b}}/{\text{L}}_{\text{c}}\times 100.$$

### Scanning Electron Microscopic (SEM) Analysis

Scanning electron microscopic (SEM) study was executed according to Kim et al. (2007) to examine the effects of CFS of *P. pentosaceus* 4I1 on the morphological changes in the cell wall of the selected pathogens, *S. aureus* KCTC-1621 and *E. coli* O157:H7. Control samples were prepared without CFS of *P. pentosaceus* 4I1. Microscopic examination was performed using a S-4300 SEM Analyzer (Hitachi, Japan).

### Determination of Growth Phase-Dependent Inhibitory Effects of CFS of 4I1

To determine whether CFS of *P. pentosaceus* 4I1 has growth phase-independent inhibitory effects, CFS of *P. pentosaceus* 4I1 at MIC + pathogenic bacteria (overnight grown single bacterial colony) were inoculated in their respective culture tubes containing 20 mL sterile NB medium followed by incubation at 37°C until 24. After every 4 h, samples were withdrawn and analyzed for the bacterial counts of pathogenic strains. Colonies were counted after growth at 37°C up to 24 h, and the log10 CFU was plotted against incubation period to prepare growth curves of individual strains. As a control, pathogenic bacterial strains without CFS of *P. Pentosaceus* 4I1 were used.

### Determination of the Effect of CFS of 4I1 on Biofilm Formation Ability of Pathogenic Strains

Pathogenic bacterial strains were inoculated and grown in NB broth and evaluated for their biofilim formation ability, and effects of CFS of *P. pentosaceus* 4I1 on biofilm formation by pathogenic bacteria was determined following the modified method of Aoudia et al. (2016). A 2 mL of bacterial cultures of *S. aureus* KCTC-1621 and *E. coli* O157:H7 (10^6^ CFU/ml) grown in NB broth were added to each glass test-tube. On the other hand, to determine the effect of CFS of *P. pentosaceus* 4I1 on biofilm formation, CFS at MIC mixed with 2 mL of bacterial cultures of *S. aureus* KCTC-1621 and *E. coli* O157:H7 (10^6^ CFU/ml) grown in NB broth and was added to each glass test-tube. While, 2 mL of NB broth was added in blank test-tubes without bacterial culture (negative control). The tubes were incubated for 48 h at 30°C. To quantify the biofilm formation, the tubes were gently washed three times with 2 mL of sterile distilled water, and attached bacteria were fixed with 2 mL of methanol for 15 min, and then, tubes were emptied and air dried at room temperature or oven dried at 60°C for 30–45 min. Subsequently, 2 mL of a 2% (v/v) crystal violet solution was added to each well and held at ambient temperature for 15 min. Excess stain was then removed by placing the test tubes under gently running tap water. A 2 mL of ethanol was used for destaining. The OD of released adherent cells was measured at 595 nm. Each assay was performed in three individual times on different days under the same conditions, and the negative control was performed in uninoculated NB broth. The cut-off OD was defined as the mean OD value of the negative control. Based on the OD, strain was confirmed for biofilm production ability in presence of CFS as a no-biofilm producer or biofilm producer (strong or week) (OD < OD of negative control confirmed as no-biofilm producer), (ODC < OD × 2 OD of negative control confirmed as week biofilm producer), moderate (2 × OD of negative control < OD ≤ 4 × OD of negative control confirmed as moderate biofilm producer) (4 × OD of negative control < OD confirmed as strong biofilm producer).

### Statistical Analysis

All experiments were performed in triplicate and results were expressed the mean ± SD following one-way ANOVA coupled with Duncan’s multiple test.

## Results

### Morphological, Biochemical, and Molecular Characterization of 4I1

**FIGURE 1:**
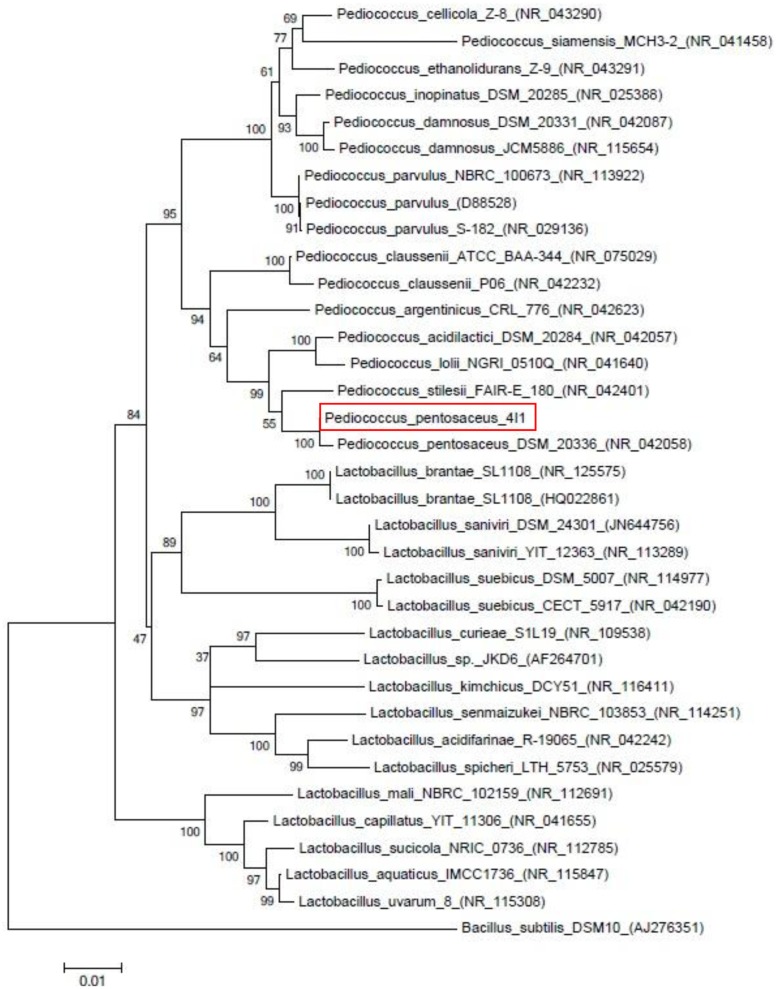
**Neighbor-joining phylogenetic tree showing the position of strain *Pediococcus pentosaceus* 4I1 among the different *Pediococcus* strains based on 16s rDNA sequences**.

Small yellow colonies of similar sizes that appeared on BCP agar using pour-plating method confirmed the presence of the LAB isolate 4I1 which was confirmed to be coccus-shaped by microscopic evaluation. Biochemical analysis of 4I1 was performed using the API 50 CHL strip kit and a selected strain was identified as a gram-positive and rod-shaped isolate (**Table 1**). API web software confirmed that strain 4I1 utilized carbohydrates, including L-arabinose, D-ribose, D-xylose, D-galactose, D-glucose, D-fructose, D-mannitol, D-sorbitol, *N*-acetylglucosamine, amygdalin, arbutin, salicin, D-cellobiose, D-maltose, D-lactose, D-melibiose, D-saccharose, D-trehalose, D-raffinose, gentiobiose, and D-turanose (**Table 1**). Color change from violet to yellow in the strip capsule indicated complete fermentation of sugar by 4I1. Molecular analysis using partial 16S rDNA gene sequencing showed the selected strain displayed 99.9% similarity with different *Pediococcus* spp. (**Figure 1**), thus, the strain was finally characterized as *P. pentosaceus* 4I1. The derived sequence was submitted to GenBank with nucleotide accession number KT372700.

**Table 1:** Biochemical characterization of *Pediococcus pentosaceus* (4I1) based on carbohydrate interpretation using API 50 CHL kit.

Active ingredient	Result	Active ingredient	Result
Glycerol	-	Salicin	+
Erythritol	-	D-cellobiose	+
D-arabinose	-	D-maltose	+
L-arabinose	+	D-lactose (bovine origin)	+
D-ribose	+	D-melibiose	+
D-xylose	+	D-saccharose	+
L-xylose	-	D-trehalose	+
D-adonitol	-	Inulin	-
Methyl-β-D-xylopyranoside	-	D-melezitose	-
D-galactose	+	D-raffinose	+
D-glucose	+	Amidon (starch)	-
D-fructose	+	Glycogen	-
D-mannose	-	Xylitol	-
L-sorbose	-	Gentiobiose	+
L-rhamnose	-	D-turanose	+
Dulcitol	-	D-lyxose	-
Inositol	-	D-tagatose	-
D-mannitol	+	D-fucose	-
D-sorbitol	-	L-fucose	-
Methyl-α-D-glucopyranoside	-	D-arabitol	-
*N*-acetylglucosamine	+	Potassium gluconate	-
Amygdalin	+	Potassium 2-ketogluconate	-
Arbutin	+	Potassium 5-ketogluconate	-
Esculin	-		


*(-): The bacterium does not use this carbohydrate; (+): The bacterium uses this carbohydrate.*

### GC–MS Analysis

The GC–MS analyses of the CFS of *P. pentosaceus* 4I1 identified seven different components accounted for 99.98% of total CFS. The CFS of *P. pentosaceus* 4I1 yielded compounds largely amino acids, organic acids, and fatty acids, as well as pyrrol derivatives which included (*S*)-2-Hydroxypropanoic acid (24.28%), caprolactam (8.83%), D-valine (28.53%), D-leucine (35.71%), 3-pyrrolidin-2-yl-propionic acid (2.43%), pyrrolo [1,2-a]pyrazine-1,4-dione, hexa (19.34%), and 9-octadecenoic acid (8.29%).

### Antibacterial Potential

**FIGURE 2:**
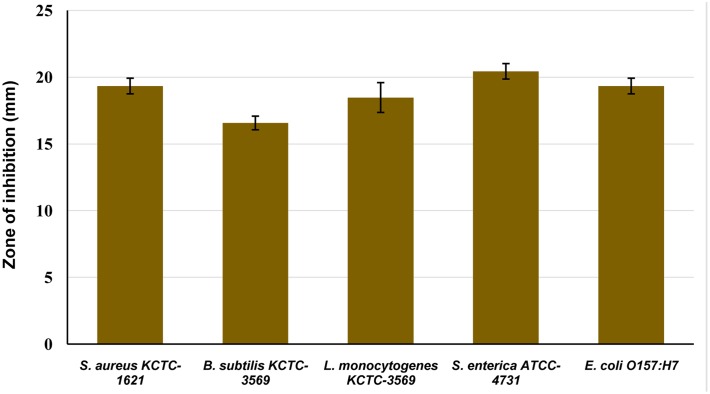
**Antibacterial activity of cell free supernatant (CFS) of *P. pentosaceus* 4I1 against foodborne pathogenic bacterial in agar well-diffusion assay.** Data are expressed as mean ± SD (*n* = 3).

The antibacterial activity of CFS of *P. pentosaceus* 4I1 against the tested foodborne pathogenic bacteria was confirmed by the presence or absence of inhibition zones on the agar well plates. As presented in **Figure 2**, CFS of *P. pentosaceus* 4I1 exhibited potent inhibitory effects against all the tested foodborne pathogenic bacteria. In this assay, *P. pentosaceus* 4I1 exerted consistent antibacterial effects against both gram-positive and gram-negative bacteria, with zone of inhibition diameters ranging from 16.5 to 20.4 mm (**Figure 2**). Sterilized distilled water and/or MRS medium used as a negative control had no inhibitory effect.

**FIGURE 3:**
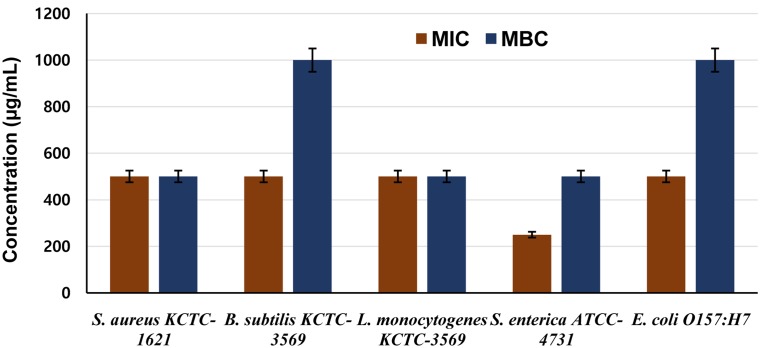
**Determination of minimum inhibitory (MIC) and minimum bactericidal concentration (MBC) concentrations of CFS of *P. pentosaceus* 4I1 against foodborne pathogenic bacteria.** Data are expressed as mean ± SD (*n* = 3).

The MIC assay revealed different susceptibilities of tested pathogens to the CFS of *P. pentosaceus* 4I1, and exhibited potent inhibitory effect as MIC and MBC values. In this assay, the MIC and MBC values of 4I1 CFS against the tested foodborne pathogens were ranged from 250 to 1,000 μg/mL (**Figure 3**). Furthermore, the CFS of 4I1 exhibited potential antibacterial effects as reflected by MIC and MBC values against all the tested pathogens (**Figure 3**). Interestingly, one of the pathogens *S. enterica* ATCC-4731 was found to be highly susceptible pathogen to the CFS of *P. pentosaceus* 4I1. Notably, both gram-positive and gram-negative bacteria were inhibited by the CFS of *P. pentosaceus* 4I1.

### Effect on Cell Viability

**FIGURE 4:**
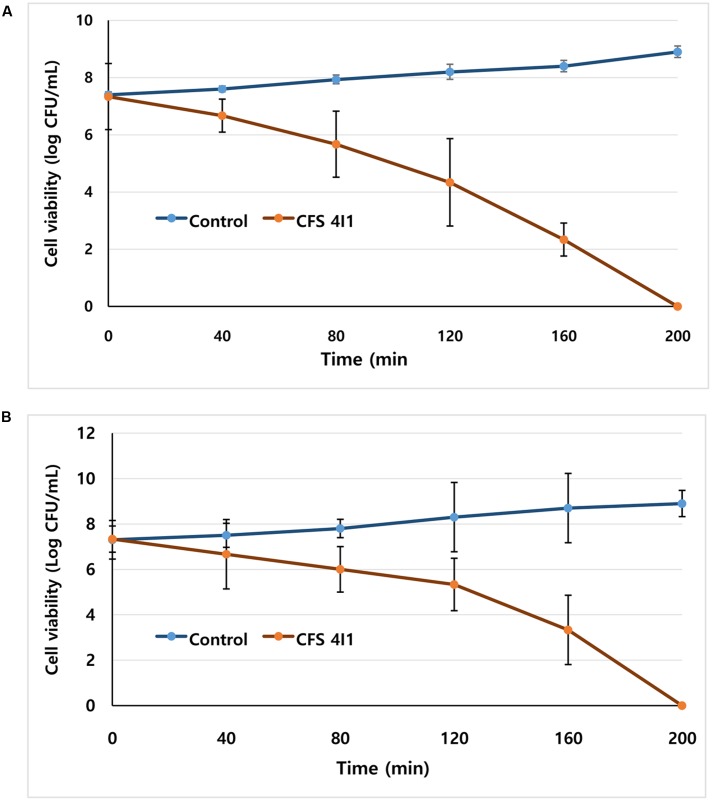
**Effect of CFS of *P. pentosaceus* 4I1 on the viability of the tested pathogenic bacteria of *Staphylococcus aureus* KCTC-1621** **(A)** and *Escherichia coli* O157:H7 **(B)**. Control without treatment. Data are expressed as mean ± SD (*n* = 3).

In this assay, the CFS of *P. pentosaceus* 4I1 when inoculated at MIC, exhibited significant inhibitory effects against the growth of tested bacterial pathogens, *S. aureus* KCTC-1621 and *E. coli* O157:H7, as confirmed by reduced bacterial cell viability when inoculated at MIC (**Figure 4**). Exposure to CFS of *P. pentosaceus* 4I1 for 0 to 80 min did not elicit severe inhibition of cell viability, but remarkable declines in the cell viable counts of *S. aureus* KCTC-1621 and *E. coli* O157:H7 was observed after exposure to the CFS of *P. pentosaceus* 4I1 for 160 min. Interestingly, the exposure to CFS of *P. pentosaceus* 4I1 for 200 min completely inhibited the cell viabilities of both tested pathogens (**Figure 4B**).

### Effect on Potassium Ion Leakage

**FIGURE 5:**
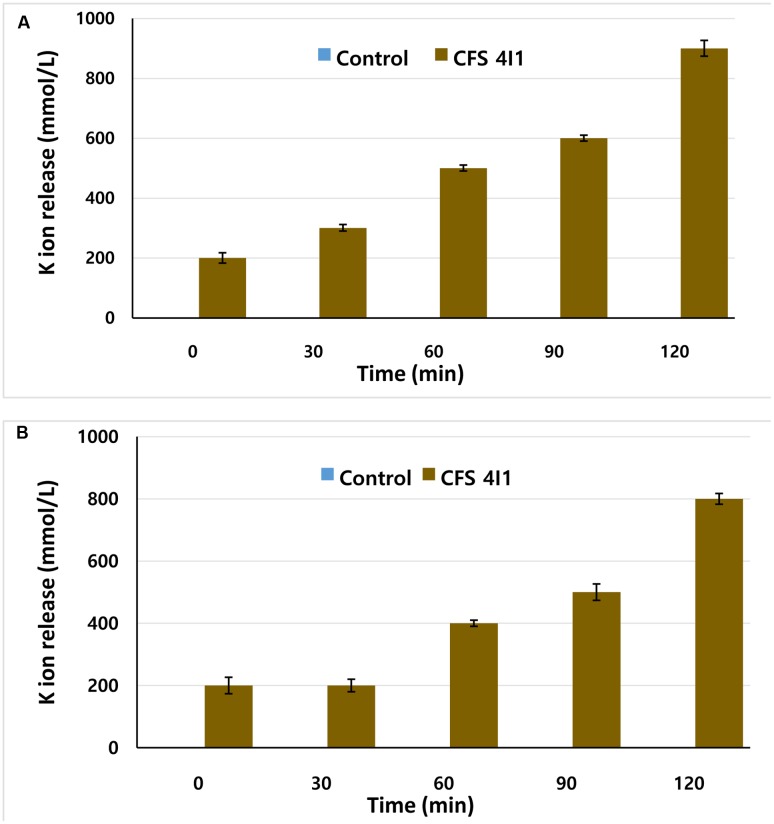
**Effect of CFS of *P. pentosaceus* 4I1 on leakage of potassium ions from the tested pathogenic bacteria of *S. aureus* KCTC-1621** **(A)** and *E. coli* O157:H7 **(B)**. Control without treatment. Data are expressed as mean ± SD (*n* = 3).

This test assay confirmed the antibacterial effect of the CFS of *P. pentosaceus* 4I1 by revealing K^+^ release from *S. aureus* KCTC-1621 and *E. coli* O157:H7 versus the control (**Figure 5**). The CFS of *P. pentosaceus* 4I1 added at MIC to cell suspensions of tested pathogenic bacteria resulted in rapid K^+^ release from the bacterial cells following protracted steady loss (**Figures 5A,B**). However, no leakage of K^+^ was observed from *S. aureus* KCTC-1621 and *E. coli* O157:H7 controls (**Figure 5**).

### Effect on Release of 260 nm Materials

**FIGURE 6:**
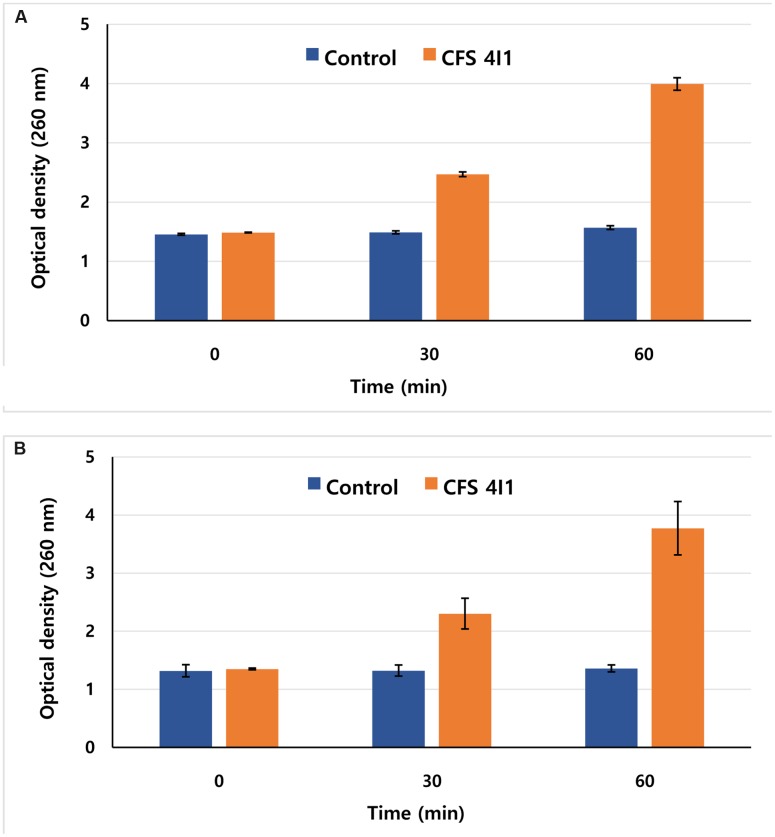
**Effect of CFS of *P. pentosaceus* 4I1 on the release rate of 260-nm absorbing material from *S. aureus* KCTC-1621** **(A)** and *E. coli* O157:H7 **(B)**. Data are expressed as mean ± SD (*n* = 3).

Release of 260-nm absorbing materials (DNA and RNA) from the cells treated with specific antimicrobials may be an indication of bacterial cell death. Hence, we evaluated the effects of the CFS of *P. pentosaceus* 4I1 on the release of 260-nm absorbing materials from *S. aureus* KCTC-1621 and *E. coli* O157:H7 treated at MIC. Interestingly, exposure of CFS of *P. pentosaceus* 4I1 to *S. aureus* KCTC-1621 and *E. coli* O157:H7 caused rapid loss of 260-nm absorbing materials from the bacterial cells. The ODs of culture filtrates of *S. aureus* KCTC-1621 and *E. coli* O157:H7 cells exposed to the CFS of *P. pentosaceus* 4I1 at 260 nm, revealed a significant time-dependent increase in the release of 260-nm-absorbing materials (**Figure 6**). However, no changes in the OD of control cells of tested pathogens were observed. Notably, exposure for 60 min to the CFS of *P. pentosaceus* 4I1 caused about a twofold increase in the OD of treated bacterial cell culture filtrates as compared with their respective controls (**Figures 6A,B**).

### Effect on Cell Membrane Permeability

**FIGURE 7:**
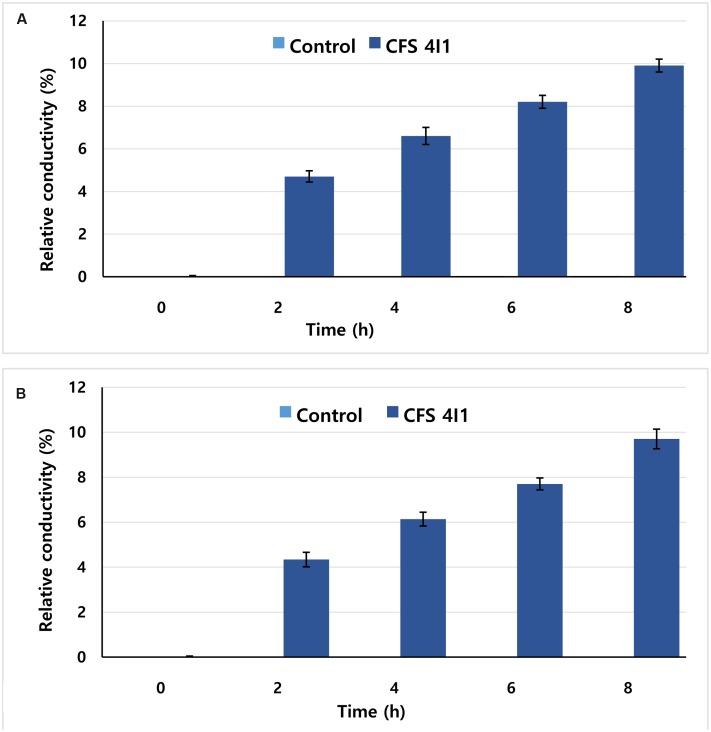
**Effect of CFS of *P. pentosaceus* 4I1 on membrane permeability of *S. aureus* KCTC-1621** **(A)** and *E. coli* O157:H7 **(B)**. Data are expressed as mean ± SD (*n* = 3).

This assay visualized the effect of the CFS of *P. pentosaceus* 4I1 at MIC on the membrane permeabilities of tested pathogens as determined by their relative electrical conductivities. As was expected, the CFS of *P. pentosaceus* 4I1 exhibited time-dependent inhibitory effect on the membrane permeabilities of the tested pathogens, and the relative electrical conductivity of each tested pathogen was increased time-dependently. Furthermore, the CFS of *P. pentosaceus* 4I1 exhibited a greater inhibitory effect on cell membrane of *S. aureus* KCTC-1621 (**Figure 7A**) than *E. coli* O157:H7 (**Figure 7B**) as indicated by their relative electrical conductivity values (**Figure 7**). No relative electrical conductivity was observed in untreated controls. In this assay, the CFS of *P. pentosaceus* 4I1 showed an ability to disrupt the plasma membranes of both tested bacteria as confirmed by the changes observed in the relative electrical conductivity values.

### Morphological Observation by SEM

**FIGURE 8:**
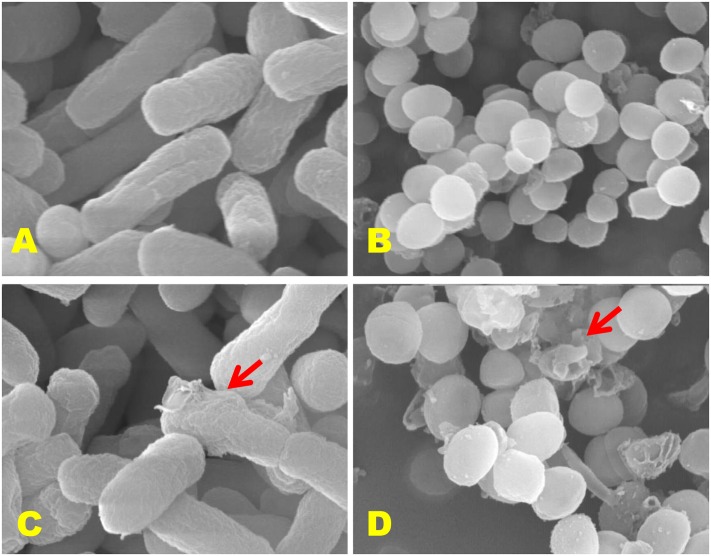
**Scanning electron microscopic (SEM) analysis of *E. coli* 0157:H7 and *S. aureus* KCTC-1621 cells treated with CFS of *P. pentosaceus* 4I1.** Controls **(A,B)** showing a regular and smooth surface; treated cells **(C,D)** arrows showing disruption and cell lysis, respectively.

Since exposure to an antimicrobial agent may lead to disruption of bacterial cell wall, we turned to SEM analysis to investigate further the effect of the CFS of *P. pentosaceus* 4I1 on the cell wall physiologies and morphologies of *S. aureus* KCTC-1621 and *E. coli* O157:H7 cells (**Figure 8**). As was expected, control bacterial cells not exposed the result of CFS of 4I1 had regular smooth surfaces (**Figures 8A,B**), whereas those treated with CFS of 4I1 at MIC showed cell wall damage and lysis (**Figures 8C,D**).

### Growth Phase-Dependent Inhibitory Effect

The growth phase-dependent behavior of CFS of *P. pentosaceus* 4I1 on the tested pathogens was measured at different time intervals as demonstrated in **Supplementary Figure S1**. In both, mono- and co-culture tubes, the lag phase for *P. pentosaceus* 4I1 lasted about 3–4 h, whereas pathogenic strains grew rapidly (**Supplementary Figure S1**). However, decreases in pathogen growths were observed in the presence of CFS of *P. pentosaceus* 4I1 after incubation for 6–12 h, and pathogen growth was reduced by almost four log cycles within 24 h of incubation as compared with the control. The effect of CFS of *P. pentosaceus* 4I1 on the growth of *S. aureus* KCTC-1621 was significantly more marked than on that of *E. coli* O157:H7.

### Effect of CFS on Biofilm Formation Ability of Pathogenic Strains

The effects of CFS of *P. pentosaceus* 4I1 on biofilim formation by *S. aureus* KCTC-1621 and *E. coli* O157:H7 were analyzed by using crystal violet assay, which showed a potent inhibitory effect of the CFS of 4I1 on the biofilm formation ability of both the tested pathogenic bacteria (**Supplementary Figure S2**). As a result, the CFS of *P. pentosaceus* 4I1 induced inhibition of biofilm formation by *S. aureus* KCTC-1621 and *E. coli* O157:H7 (**Supplementary Figure S2**) as confirmed by a significant (*p* < 0.05) reduction in OD values, while in control without CFS of *P. pentosaceus* 4I1, *S. aureus* KCTC-1621 and *E. coli* O157:H7 showed a strong biofilm formation ability (**Supplementary Figure S2**). Our results indicate that CFS of *P. pentosaceus* 4I1 has potential effect against biofilm formation ability of *S. aureus* KCTC-1621 and *E. coli* O157:H7.

## Discussion

In this study, we characterized a lactic acid bacterium 4I1 isolated from freshwater fish *Zacco koreanus* as *P. pentosaceus* 4I1 based on its morphological, biochemical, and molecular characteristics (Bajpai et al., 2016b; Casaburi et al., 2016). Furthermore, LAB isolate 4I1 showed remarkable antibacterial activities against a panel of foodborne pathogens as confirmed by inhibitory zones in the agar well-diffusion assay and different susceptibilities in the MIC/MBC assay. As reported previously, other LAB have shown potential antibacterial effects against a number of foodborne pathogens (Amenu, 2013). Furthermore, a variety of pathogenic and foodborne pathogenic bacteria exhibit susceptibility to LAB (Tadesse et al., 2005; Carina Audisio et al., 2011; Darsanaki et al., 2012; Yah et al., 2014). In subsequent assays, we randomly selected two foodborne pathogenic bacteria, *S. aureus* KCTC-1621 and *E. coli* O157:H7, to evaluate the antibacterial effects of CFS of *P. pentosaceus* 4I1.

This study shows exposure to CFS of 4I1 reduces *S. aureus* KCTC-1621 and *E. coli* O157:H7 viable cell counts. Previous reports have confirmed the inhibitory effects of various LAB strains on the viabilities of foodborne pathogenic bacteria (de Barros et al., 2012). Recently, we also reported that the CFS of lactic acid bacterium isolated from Kimchi (a Korean fermented food) exhibits inhibitory effects (Bajpai et al., 2016b). Other LAB have also been shown to exhibit antibacterial effects with respect to inhibiting the cell viabilities of foodborne pathogenic bacteria (Carina Audisio et al., 2011).

The bacterial plasma membrane plays an important barrier function, for example, it inhibits the transit of various important electrolytes, such as, the potassium ion, which participates in various cell membrane functions and in essential enzymatic activities. The regulation of these important electrolytes is critical, for example, elevated leakage of potassium ion can cause bacterial cell membrane disruption, and thus, it is necessary to maintain the equilibrium of essential ions in order to maintain energy status and survival (Cox et al., 2001). Changes in the structural integrity of the bacterial cell membrane may increase potassium ion release, and abrupt cell metabolism, thus induce cell death (Cox et al., 2001). In a previous study, inhibitory effects of CFS derived from other LAB were confirmed to involve potassium ion efflux (Bajpai et al., 2016b).

In the present study, it was observed the CFS of *P. pentosaceus* 4I1 at MIC had remarkable effects on the release of 260 nm materials (DNA and RNA) from cells of tested pathogenic bacteria, which confirmed its potential role as a potent antibacterial agent. Marked release of 260-nm materials from CFS-treated cells of pathogenic bacteria was supported by observations of loss of cell membrane structural integrity, which would lead to the loss of essential cell electrolytes (Farag et al., 1989). These observations suggest that the release of 260-nm absorbing materials from *S. aureus* KCTC-1621 and *E. coli* O157:H7 might provide sensitive indicators of membrane damage and loss of membrane integrity. Similar results on the inhibitory effect of CFSs or LAB on nucleic acid release from bacterial pathogens have been previously reported (Alakomi et al., 2000; Bajpai et al., 2016b).

In this study, SEM analysis showed marked morphological changes to the cell walls of *S. aureus* KCTC-1621 and *E. coli* O157:H7 resulting in cell wall deformation by the CFS of 4I1. Consistent with our findings, other LAB isolates have also been demonstrated to induce such morphological alterations in several pathogenic microbes (Kim et al., 2009). These morphological alterations may be due to aberrations in membrane lipid composition, altered membrane fluidity and/or membrane integrity resulting in cell wall lysis and loss of intracellular dense material (Sikkema et al., 1994).

Changes in relative electrical conductivity can adversely affect membrane integrity and eventually result in cell death, and thus, the maintenance of bacterial cell membrane integrity is required to secure normal cell metabolism (Cox et al., 2001). Our results suggest CFS of *P. pentosaceus* 4I1 severely disrupted the plasma membranes of tested foodborne pathogens and that this resulted in increased loss of essential metabolites and necessary ions. These findings agree well with those of Roth and Keenan (1971), who reported that LAB have the ability to cause sub-lethal injury to *E. coli*. In addition, similar results have also been reported for LAB derived organic acids, such as, acetic acid (Przybylski and Witter, 1979). Furthermore, their effects were attributed to disruption of the lipopolysaccharide layer (Przybylski and Witter, 1979). Similarly, LAB-derived CFSs and/or other antimicrobials have also been shown to markedly affect the relative electrical conductivity parameters of foodborne pathogens (Patra et al., 2015; Bajpai et al., 2016b).

This study also revealed growth phase-dependent inhibitory effects of the CFS of *P. pentosaceus* 4I1. The inhibitory effect observed for the tested pathogens in growth-phase dependent inhibition assay began from the early stationary phase, which could be mediated by secondary metabolites, organic acids, or other compounds produced in the CFS of *P. pentosaceus* 4I1. Similar results were observed by Muhsin et al. (2015).

Moreover, the antimicrobial activities of producer strains may be related to the action of various compounds, such as, organic acids, H_2_O_2_, and bacteriocin-like substances (Anas et al., 2008). A number of reports have confirmed strains of *Lactobacilli* are not able to regenerate hydrogen peroxide under anaerobic conditions, and that antimicrobial activities are unaffected by acidity (Juillard et al., 1987). Furthermore, most *Lactobacilli* strains produce bacteriocins or bacteriocin-like substances with wide antimicrobial spectrums (Klaenhammer, 1988; Piard and Desmazeand, 1991; Nettles and Barefoot, 1993). During our preliminary screening, we treated CFS of 4I1 with trypsin (a proteolytic enzyme) and found lost its activity, indicating the antimicrobial activity of *P. pentosaceus* 4I1 might be due to the production of proteinous substances, such as, bacteriocin-like substances, as was previously reported (Ghrairi et al., 2008).

Another concern regarding H_2_O_2_-related antimicrobial activity, according to Prado et al. (2000), is that lyophilization promotes the removal of oxygen metabolites and H_2_O_2_. Rodriguez et al. (1997) emphasized H_2_O_2_ is rapidly degraded in the MRS broth, which excludes the possibility that the antimicrobial activity demonstrated by lyophilized *Lactobacilli* involves H_2_O_2_, as in this study, MRS was used as the growth medium for the test producer strain and lyophilization was used to concentrate the supernatant for later resuspension. de Oliveira et al. (2014) also reported similar findings for lyophilized cultures.

The results of the present study indicate the inhibitory effect of CFS of 4I1 might be due to the actions of organic acids produced by LAB strains. The lyophilized concentrated supernatant from strain 4I1 was found to be sensitive to neutralization with 4 M NaOH solution, and 24, 48, and/or 72 h after neutralization, it completely lost its inhibitory ability against tested pathogenic strains (data not shown). Thus, we conclude the antimicrobial activities observed in the present study involved the actions of organic acids and/or the acidification of medium. Similar results were presented by Pereira and Gómez (2007), who evaluated the antimicrobial potential of a commercial *Lactobacillus acidophilus* strain on foodborne pathogens (*E. coli* and *S. aureus*). Organic acids contribute to the control of microorganisms and reduce food pH values, which adversely affects the survival and proliferation of pathogenic microbes, including gram-positive and gram-negative bacteria (Tamanini et al., 2012).

To confirm that the antimicrobial action of CFS of 4I1 was organic acid mediated, we subjected CFS of 4I1 to GC–MS analysis. The obtained confirmed the presence of fatty acids and other organic acids in the CFS of 4I1, importantly responsible for antimicrobial properties of *Lactobacilli* strains. Based on the above, we hypothesized that the antimicrobial activity of 4I1 observed in this study might be mediated by its production of bacteriocin-like substances and/or organic acids. However, the synergistic effects of acids, hydrogen peroxide, and bacteriocin-like substances cannot be ruled out (Anas et al., 2008). Sjögren et al. (2003) also confirmed the antimicrobial properties of 3-(R)-hydroxydecanoic acid, 3-hydroxy-5-*cis*-dodecenoic acid, 3-(R)-hydroxydodecanoic acid, and 3-(R)-hydroxytetradecanoic acid, from *Lactobacillus plantarum* MiLAB 14, and Sharma et al. (2014) confirmed the presence of octadecanoic acid in *Lactobacillus helveticus* by GC–MS. Haque et al. (2009) reported that of the organic acids examined, propionic acid (PA) was an effective antimicrobial, and thus, further supported the organic acid-mediated inhibitory effect of *Lactobacilli* strains, and the outcome of the present study.

Under adverse conditions, many pathogenic bacteria have the ability to produce biofilms (Kim et al., 2013), which protect cells by restricting the access of antimicrobials, and thus, biofilm-forming bacteria pose a substantial threat to human health. In the present study, CFS of *P. pentosaceus* 4I1 inhibited biofilm formation by *S. aureus* KCTC-1621 and *E. coli* O157:H7. Woo and Ahn (2013) reported similar results for probiotic strains against *L. monocytogenes* and *Salmonella*. Similarly, Kim et al. (2013) found that *L. acidophilus* A4 exhibited strong anti-biofilm activity against the growth of *E. coli* O157: H7, *Salmonella enteritidis*, *Salmonella typhimurium* KKCCM11806, *Yersinia enterocolitica*, *Pseudomonas aeruginosa* KCCM 11321, *L. monocytogenes*, and *Bacillus cereus*. Also, it has been reported that LAB-derived CFSs produce antimicrobial molecules that have significant potential to inhibit foodborne pathogenic bacteria such as *E. coli* O157:H7, *S. enterica* serovar Enteritidis, *S. aureus* and *L. monocytogenes* (Aoudia et al., 2016).

This study shows the newly identified lactic acid bacterium *P. pentosaceus* 4I1, which was isolated from the intestinal microbiota of the freshwater fish *Zacco koreanus*, has marked inhibitory effects against foodborne pathogenic bacteria in different *in vitro* models. More specifically, the CFS of *P. pentosaceus* 4I1 had a significant inhibitory effect on membrane permeability, as reflected by reduced cell viability and the cellular release of 260-nm absorbing materials and potassium ions, and increased relative conductivity. Morphological alterations (observed by SEM) and protective biofilm formation ability further supported potent antibacterial activity of *P. pentosaceus* 4I1. These findings reinforce suggestions that *P. pentosaceus* 4I1 could be used as an effective antimicrobial agent against foodborne pathogens.

## Author Contributions

VB, IR, JL, J-IY performed experiments and drafted manuscript; VB, IR, J-HH contributed interpretation, analyzed data and wrote paper, CP, JL, WP, Y-HP contributed for conception, designed experiment, analyzed data, and provided technical support.

## Conflict of Interest Statement

The authors declare that the research was conducted in the absence of any commercial or financial relationships that could be construed as a potential conflict of interest.
