# Supplementary material for: Characterization and Antibacterial Potential of Lactic Acid Bacterium Pediococcus pentosaceus 4I1 Isolated from Freshwater Fish Zacco koreanus
Source: Front Microbiol. 2016 Dec 20;7:2037. doi: 10.3389/fmicb.2016.02037 (PMC5167689; doi:10.3389/fmicb.2016.02037)
Supplement: Supplementary file 1 [file Data_Sheet_1.DOCX]

**Characterization and Antibacterial Potential of Lactic Acid Bacterium *Pediococcus pentosaceus* 4I1 Isolated from Freshwater Fish *Zacco koreanus***

***Vivek K. Bajpai^1,$^, Jeong-Ho Han^2,$^, Irfan A. Rather^1,*^, Chanseo Park^2^, Jeongheui Lim^2,*^, Woon Kee Paek^2^, Jong Sung Lee^3^, Jung-In Yoon^3^* *and Yong-Ha Park^1,^****

*^1^ Department of Applied Microbiology and Biotechnology, Yeungnam University, Gyeongsan, Gyeongbuk 712-749, Republic of Korea*

*^2^ National Science Museum, Ministry of Science, ICT and Future Planning, Daejeon, 305-705, Republic of Korea*

*^3^Kcellbio, Seoulsoop Kolon Digital Tower, Seongsuil-ro-4-gil, Seongdong-gu 04713, Seoul, Korea*

**^$^ Both authors contributed equally**

**Running head:** Antibacterial action of *P. pentosaceus* 4I1

To whom correspondence should be addressed:

**Prof. (Dr.) Yong-Ha Park (Correspondence)**

E-mail: peter@ynu.ac.kr; Fax: +82-53-813-4620

**Dr.** **Irfan A. Rather (Co-correspondence)**

E-mail: erfaan21@gmail.com; Fax: +82-53-813-4620

**Dr.** **Jeongheui Lim (Co-correspondence)**

E-mail: jhlim1226@naver.com; Fax: +82-42-601-7788

**Supplementary Data**

**FIGURE S1** Growth phase-dependent antibacterial effect of the CFS of *P. pentosaceus* 4I1 against *S. aureus* KCTC-1621 and *E. coli* O157:H7.


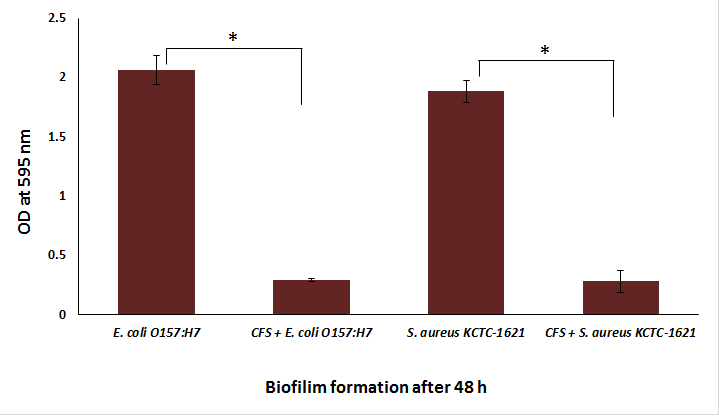


**FIGURE S2** Effect of cell free supernatant (CFS) of *P. pentosaceus* 4I1 on biofilm formation by *S. aureus* KCTC-1621 and *E. coli* O157:H7. * Significantly different at p<0.05 by Duncan’s multiple range test.
